# Early real-world experience using temperature-guided diamond tip facilitated high-power ablation for catheter ablation of atrial fibrillation

**DOI:** 10.1007/s10840-023-01505-z

**Published:** 2023-02-14

**Authors:** Sebastian Dittrich, Martin Braun, Leonard Bergau, Christian Sohns, Arian Sultan, Jakob Lüker, Jonas Wörmann, Cornelia Scheurlen, Jan-Hendrik Schipper, Jan-Hendrik van den Bruck, Karlo Filipovic, Philipp Sommer, Daniel Steven

**Affiliations:** 1 Department of Electrophysiology, University Hospital Cologne, University of Cologne, Kerpener Str. 62, 59037 Cologne, Germany; 2 Clinic for Electrophysiology, Herz- Und Diabeteszentrum NRW, Bad Oeynhausen, Germany

## Purpose

The accepted standard for radiofrequency (RF) pulmonary vein isolation (PVI) today is performing a point-by-point, antral, wide circumferential isolation of the pulmonary veins, using a three-dimensional mapping system [1]. However, conventional point-by-point RF PVI aiming for transmural lesion creation is a time-consuming procedure and long-term success after PVI is significantly related to reconduction [2]. With use of conventional RF catheters, lesion gaps leading to AF recurrence are still occurring frequently [3]. The DiamondTemp® (DT) RF catheter features six thermocouples at its tip, imbedded in an industrial diamond-like material which offers high thermal diffusivity. It allows for temperature-guided ablation, providing high ablation power with short RF durations (HPSD) and direct temperature monitoring, aiming for safe and transmural lesion formation. We provide real-world observational data for *de novo* and repeat AF ablation procedures as well as long-term follow-up, including mapping results of re-do procedures.

## Methods

We retrospectively analyzed procedural data from 80 patients who underwent *de novo* PVI or repeat AF ablation. In 40 patients, AF ablation using the DT catheter was performed. A matched control group of 40 patients received ablation using a standard, force-sensing ablation catheter.

### Ablation procedure

In all *de novo* PVI procedures, a paired, antral, ipsilateral pulmonary vein isolation was performed in a point-by-point fashion. In case of the DT catheter, a temperature-controlled mode was used with a maximum tolerated temperature of 60 °C. Maximum output power was set to 50 W. In case of the ST catheter, a power-controlled mode was set to a maximum of 40 W while applying RF lesions to the anterior wall of the left atrium and 30 W for the posterior wall, guided by ablation index. In repeat procedures, ablations beyond PVI were carried out at operator’s discretion including repeat PVI alone, ablation of complex fractionated electrograms, application of lines, or posterior wall isolation.

### Follow-up

Follow-up of patients was acquired 3 months and 1 year post ablation procedure. Patients were either seen by a rhythmologist in an outpatient clinic or contacted by phone. Patients were asked to provide a subjective assessment of recurrence or a 24-h Holter ECG. Patients who reported symptoms of arrhythmia recurrence on the phone were scheduled for a Holter ECG. Patients with devices (pacemaker, ICD, event recorder) were subject to device interrogation. Events in the blanking period (until 90 days post ablation procedure) were excluded.

### Statistics

Statistical analysis was performed using SPSS, R Studio software, and GraphPad Prism. Matching of control group was performed using a propensity score based, bipartite matching method, matching for comorbidities and type of procedure performed (PVI, additional CTI).

## Results

### Procedure data for *de novo* PVIs

**Fig. 1:**
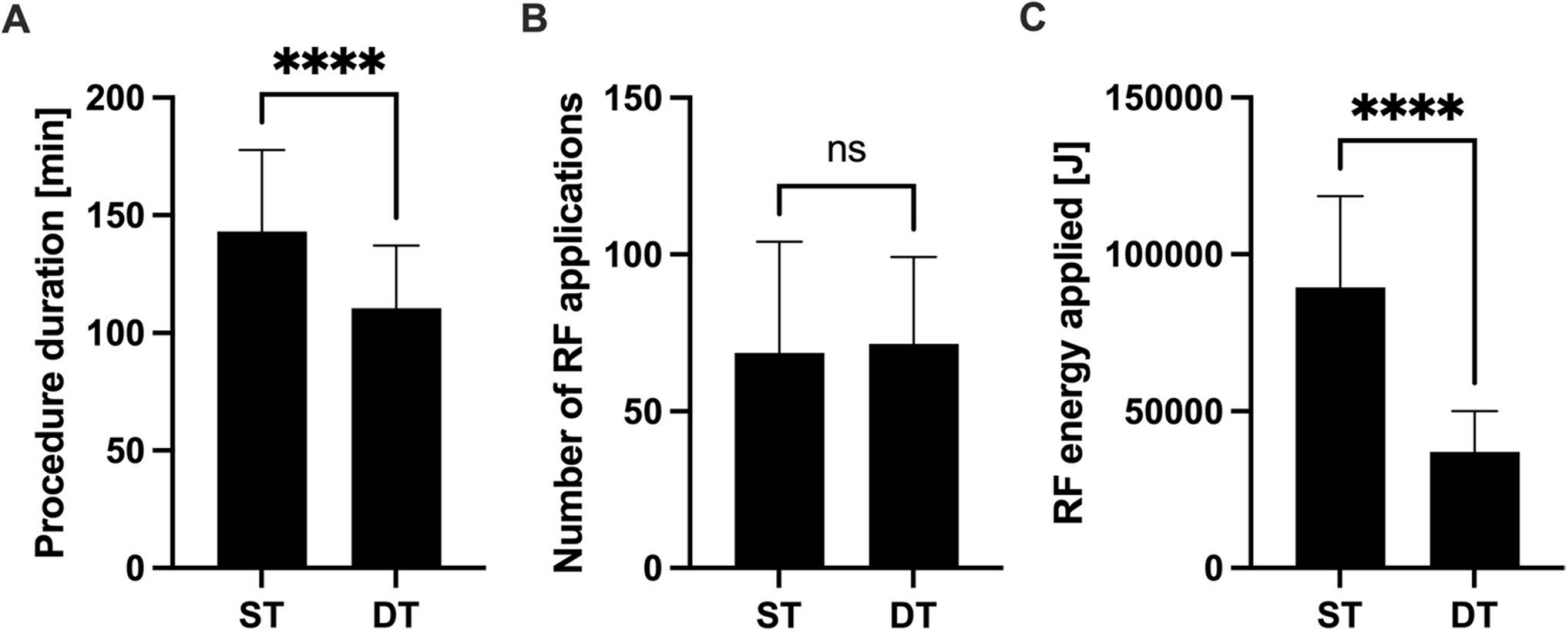
Procedural data for *de novo* procedures. The bar graphs display procedure durations (**A**), number of applied RF applications (**B**), and total applied RF energy (**C**) in the ST and DT groups (****, *p* > 0.001; ns, non-significant)

Procedure data for *de novo* procedures are displayed in Fig. 1. DT-guided AF ablation resulted in shorter procedure duration (110.4 min vs. 144.5 min, *p* < 0.02) and reduced applied RF energy (37,097 J vs. 87,396 J, *p* < 0.02). The number of RF applications needed for completion of PVI did not differ significantly between the two groups, with 71.5 using the DT ablation catheter versus 68.7 using the ST ablation catheter (*p* = 0.73).

### Long-term follow-up for *de novo* procedures

In 55 out of 62 patients (87%), a 1-year follow-up was available, while 6 patients (9.7%) were lost to follow-up and one patient in the ST group had only been scheduled for a 3-month follow-up at the time of submission.

In the DT group, less patients presented with arrhythmia recurrence compared to the ST group, without reaching statistical significance (5/28, 16.1% vs. 10/27, 37%; *p* = 0.14). Entities of arrhythmia recurrence consisted of paroxysmal AF (DT: 4/5; ST: 4/10), persistent AF (DT: 0/5; ST: 4/10), and atrial flutter (DT: 1/5; ST: 2/10). Re-do procedures were scheduled for 2 patients in the DT group and 4 in the ST group.

Mapping data of re-do procedures were available for one patient in the DT group and showed reconnection of left pulmonary veins. In the ST group, mapping data of re-do procedures was available for 4 patients and included reconnection of the right pulmonary veins in two cases, reconnection of all pulmonary veins in one case, and reconnection of the right pulmonary veins and left superior pulmonary vein in one case.

### Procedure data for repeat AF ablations

Eighteen patients underwent repeat AF ablation, evenly distributed between both groups. Among these patients, no significant differences could be observed in procedure duration (177.8 min vs. 180 min, *p* = 0.90) and number of RF applications (105.1 vs. 122.1, *p* = 0.57), with significantly less applied RF energy in the DT group (68,432 J vs. 121,132 J, *p* = 0.023).

### Safety

No procedure-related complications requiring intervention occurred. In one patient, single mispuncture during LA access was reported in the DT group without further sequela. DT ablation did not reveal higher esophageal temperatures during procedures than ST ablation.

## Discussion

In the present manuscript, we provide early observational experience of patients that underwent PVI and repeat AF ablation with the DT ablation catheter, including mapping data of re-ablation procedures in case of arrhythmia recurrence. Usage of the novel catheter significantly reduced procedure times of *de novo* pulmonary vein isolations. These results are in line with the recently published papers by Kautzner et al. [4], Starek et al. [5], and Neuzil et al. [6] and expectations for an ablation approach that uses higher energy outputs.

Importantly, long-term follow-up did not reveal higher rates of arrhythmia occurrence in the DT group and rates of PV reconnection in the DT group were not higher in our collective than in the ST group. However, small sample size limits the value of these findings. Our observations are in line with other studies evaluating HPSD strategies that indicate reconnection rates are not higher than those of conventional strategies [7].

In case of repeat AF ablation procedures, DT usage was feasible safe in our cohort. Due to small sample size and heterogeneity of performed ablations, generalization is limited.

## Conclusion

In our clinical experience, AF ablation using the DT catheter resulted in shorter procedure durations for *de novo* PVI, applying a lower amount of RF energy, compared to a standard force-sensing catheter. Intraprocedural complications were comparably low in both groups. In long-term follow-up, no significant differences could be observed between DT and ST regarding arrhythmia recurrence and PV reconnection.

## Data Availability

All data and material are available upon contacting the corresponding author.
